# Differences in Density Dependence among Tree Mycorrhizal Types Affect Tree Species Diversity and Relative Growth Rates

**DOI:** 10.3390/plants11182340

**Published:** 2022-09-07

**Authors:** Boliang Wei, Lei Zhong, Jinliang Liu, Fangdong Zheng, Yi Jin, Yuchu Xie, Zupei Lei, Guochun Shen, Mingjian Yu

**Affiliations:** 1 College of Life Sciences, Zhejiang University, Hangzhou 310058, China; 2 Zhejiang Wuyanling National Nature Reserve Management Bureau, Taishun 325500, China; 3 College of Life and Environmental Science, Wenzhou University, Wenzhou 325035, China; 4 State Key Laboratory of Plant Physiology and Development in Guizhou Province, School of Life Sciences, Guizhou Normal University, Guiyang 550025, China; 5 Zhejiang Tiantong Forest Ecosystem National Observation and Research Station, Center for Global Change and Ecological Forecasting, School of Ecological and Environmental Sciences, East China Normal University, Shanghai 200241, China

**Keywords:** conspecific negative density dependence, tree mycorrhizal types, heterospecific mycorrhizal tree neighbors, species diversity, relative growth rate, forest carbon sink rates

## Abstract

Conspecific negative density dependence (CNDD) may vary by tree mycorrhizal type. However, whether arbuscular mycorrhizal (AM)-associated tree species suffer from stronger CNDD than ectomycorrhizal (EcM) and ericoid mycorrhizal (ErM)-associated tree species at different tree life stages, and whether EcM tree species can promote AM and ErM saplings and adults growth, remain to be studied. Based on the subtropical evergreen broad-leaved forest data in eastern China, the generalized linear mixed-effects model was used to analyze the effects of the conspecific density and heterospecific density grouped by symbiont mycorrhizal type on different tree life stages of different tree mycorrhizal types. The results showed that compared to other tree mycorrhizal types at the same growth stage, EcM saplings and AM adults experienced stronger CNDD. Heterospecific EcM density had a stronger positive effect on AM and ErM individuals. Species diversity and average relative growth rate (RGR) first increased and then decreased with increasing basal area (BA) ratios of EcM to AM tree species. These results suggested that the stronger CNDD of EcM saplings and AM adults favored local species diversity over other tree mycorrhizal types. The EcM tree species better facilitated the growth of AM and ErM tree species in the neighborhood, increasing the forest carbon sink rate. Interestingly, species diversity and average RGR decreased when EcM or AM tree species predominated. Therefore, our study highlights that manipulating the BA ratio of EcM to AM tree species will play a nonnegligible role in maintaining biodiversity and increasing forest carbon sink rates.

## 1. Introduction

Biodiversity is rapidly decreasing due to human impacts [1,2,3,4]. The release of large amounts of CO_2_ exacerbates the greenhouse effect and further accelerates the extinction of certain species [5,6,7,8]. As important ecosystems on earth, forests play an important role in maintaining species diversity and carbon sequestration and storage. However, research finds that forest productivity declines as species diversity declines [9,10,11]. Therefore, in the context of global change, protecting and improving tree species diversity is crucial in increasing the forest productivity and slowing down the rate of species extinction.

Ecologists have proposed a plethora of theories and hypotheses to explain the mechanisms by which species diversity is maintained [12,13,14,15,16]. Among them, conspecific negative density dependence (CNDD) is an ecological process in which individuals have increased mortality and slowed growth rates surrounded by high-density conspecific neighbors due to the accumulation of host-specific natural enemies, thus promoting the coexistence of diverse species [17,18,19,20]. Research shows that symbiotic fungi that form mycorrhizae with plant roots and soil-borne pathogens that cause plant disease can play important but distinct roles in CNDD [21,22,23,24].

Mycorrhizal fungi play an important role in maintaining plant diversity and improving ecosystem function [24,25]. These symbiotic fungi provide up to 80% of the plant's needs for nitrogen and phosphorus to the host plant in exchange for carbohydrates [26,27]. Almost all woody plants form symbiotic relationships with arbuscular mycorrhizal (AM), ectomycorrhizal (EcM), or ericoid mycorrhizal (ErM) fungi [28], which we refer to as AM, EcM, or ErM tree species. Previous studies have found that symbiotic fungi attenuate the strength of CNDD and improve individual survival by promoting plant nutrient acquisition and resistance to soil-borne pathogens [29,30]. Furthermore, trees associated with different mycorrhizal fungi have different capacities for plant nutrient uptake and pathogen defense [27,31,32,33]. Therefore, different tree mycorrhizal types can play different roles in regulating species diversity and regulating forest carbon sink rates.

Since AM tree species usually experience stronger CNDD than EcM tree species, species diversity around AM tree species is usually higher [34,35], while EcM tree species generally maintain low-diversity, monodominant forests [36,37]. However, Qin et al. [38] found that AM saplings had stronger CNDD than EcM saplings, while AM juveniles had the same CNDD as EcM juveniles. This means that the CNDD of different tree mycorrhizal types varies with tree life stage. However, it is unclear whether this variation affects species diversity.

Unlike the negative effects of conspecific individuals, the existence of heterospecific neighbors alleviates the strength of CNDD and promotes the survival of focal individuals due to herd immunity effects [39,40,41]. Since the strength and direction of impacts vary by tree-mycorrhizal-type neighbors, mixing all heterospecific individuals together would overlook the different effects of these heterospecific mycorrhizal tree neighbors [34,42,43]. Therefore, it is necessary to group heterospecific species according to mycorrhizal symbionts. While neighborhood EcM tree species can improve seedling survival compared to AM tree species [29], it is unclear how different heterospecific mycorrhizal tree neighbors affect the growth of saplings and adults, and whether this influence will affect the forest carbon sink rate.

Exploring the differences in CNDD among AM, EcM and ErM tree species at sapling and adult stages will help to reveal the role of mycorrhizal associations in maintaining species diversity and regulating forest carbon sink rates. Here, we obtained two census data from a 9 ha (300 m × 300 m) subtropical forest dynamic plot in Wuyanling National Nature Reserve, Zhejiang Province, eastern China, to study the effects of neighborhood factors (conspecific density and heterospecific density grouped by symbiont mycorrhizal types) on the survival and growth of different tree mycorrhizal types along tree life stages. We aimed to explore the following questions: (1) Do AM tree species suffer from stronger CNDD than EcM or ErM tree species along tree life stages? (2) Do neighborhood EcM tree species promote the growth and survival of different tree mycorrhizal types at different tree life stages? (3) How does the variance in the strength of CNDD in different tree mycorrhizal types affect species diversity and forest carbon sink rates?

## 2. Methods

### 2.1. Study Site

This study was conducted in Wuyanling National Natural Reserve (119°37′08″–119°50′00″ E, 27°20′52″–27°48′39″ N), Taishun County, Zhejiang Province, eastern China. The reserve is approximately 18,861.5 ha. The mean annual temperature is 15.2 °C. The mean annual precipitation is 2195.8 mm, mostly between May and June. According to the records of the Shangfengxiang Meteorological Station (1040 m above sea level) near the study site, the mean annual temperature in the nearby area is 14.0 °C, with the lowest mean monthly temperature in January (4.0 °C) and the highest in July (23.0 °C), and the extreme lowest temperature is −8.9 ℃. The frost period begins in early October and ends in early April of the following year. Frost-free days are about 210 days, and the sunshine rate is 38% [44].

**Figure 1:**
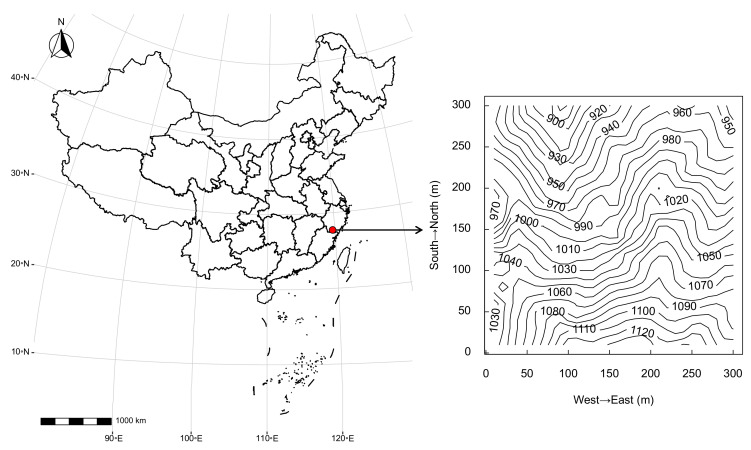
Location and contour map of the 9 ha forest dynamic plot in Wuyanling National Natural Reserve, eastern China.

In 2013, we established a 9 ha (300 m × 300 m) forest dynamic plot (119°40′13.73″ E, 27°42′20.27″ N) in an evergreen broad-leaved forest in the reserve (Figure 1). All trees with diameter at breast height (DBH, 1.3 m) ≥ 1 cm were tagged, identified to the species level, mapped and measured according to standard CTFS-ForestGEO protocols [45]. The second census was completed in 2018. The plot is 869 m to 1144 m above sea level. According to the second census, there were 63,158 free-standing woody plant individuals with DBH ≥ 1 cm in the plot, belonging to 52 families, 94 genera and 192 species. The dominant canopy species are *Castanopsis eyrei* (Fagaceae), *Cyclobalanopsis stewardiana* (Fagaceae) and *Schima superba* (Theaceae).

### 2.2. Focal Species and Mycorrhizal Associations

We assigned each individual to one of two life stages (saplings or adults) according to LaManna et al. [46], Liu et al. [47] and Pu and Jin [48] in subtropical forests. Saplings were defined as individuals with 1 cm ≤ DBH < 2 cm for shrubs, 1 cm ≤ DBH < 5 cm for understory tree species, and 1 cm ≤ DBH < 10 cm for canopy tree species. Individuals with DBH larger than a sapling were defined as adults. The life forms of these species were classified according to the Flora of China [49] and the Flora of Zhejiang [50] (Table S1). For this study, we focused on the census between 2013 and 2018. Survival information was recorded as 1 if the individual was alive and 0 if the individual was dead. We calculated the relative growth rate (RGR) for each individual in the 5-year census interval from 2013 to 2018. RGR was calculated as (log(*BA_t_*_+Δ*t*_) − log(*BA_t_*))/Δ*t*, where *BA* indicates the sum of the basal area (BA) of an individual at successive time steps *t*.

Mycorrhizal types of plant species were determined according to published literature and the FungalRoot data set [51]. In the absence of information on the mycorrhizal type of a given species, we referred to the mycorrhizal type of its congeners (Table S1) [52]. In total, we obtained 146 arbuscular mycorrhizal (AM) species, 24 ectomycorrhizal (EcM) species and 9 ericoid mycorrhizal (ErM) species (Table 1).

### 2.3. Neighborhood Factors

Four neighborhood factors (NF) were calculated for each focal individual: density of conspecific neighbors (Con), density of heterospecific AM neighbors (HetAM), density of heterospecific EcM neighbors (HetEcM), density of heterospecific ErM neighbors (HetErM). NF was defined as the distance-weighted (Dist) sum of the BAs of conspecific or heterospecific neighbors found within a certain radius (*r*) of each focal individual, divided by the circular area (π*r*^2^). To account for the potentially nonlinear nature of local biotic interactions, we introduced the exponent *c* as Equation (20) in Detto et al. [53] to calculate NF, where we set 10 *c* from 0.1 to 1, and selected the *c* value with the maximum likelihood value (Figures S1 and S2 and Table S2). NF was calculated as:

(1) $${\mathrm{NF}}_{i}={\left(\frac{1}{\pi r^{2}}\overset{n}{\underset{j=1}{\sum }}\frac{BA_{j}}{Dist_{ij}}\right)}^{c}$$

where *n* is the number of neighbors within radius *r*, *BA_j_* is the basal area of neighbor *j*, *Dist_ij_* is the distance between focal individual *i* and its neighbor *j*.

### 2.4. Statistical Analyses

We used generalized linear mixed-effects models (GLMMs) [54] with a binomial error distribution to quantify the effect of local neighbors on individual survival probability. We used linear mixed-effects models (LMMs) [55] to assess the influence of neighborhood density on individual RGR. The fixed effects of models included log-transformed individual size (i.e., DBH measured during the first census) and four scale-dependent neighborhood factors (Con, HetAM, HetEcM, and HetErM). To account for spatial autocorrelation and interspecific differences, quadrat (20 m × 20 m subplots) and species identity of focal individuals were considered as random effects in the model [56]. Since different species have different growth rates and may exhibit different relationships between size and survival, we allowed the effect of initial size to vary by species (i.e., random slopes) [41]. The model was summarized as follows:

Survival*_ij_* ~ Binomial(*p_ij_*) (2)

RGR*_ij_* ~ N(*λ_ij_*, *σ*^2^*_λ_*) (3)

Logit (*p_ij_*) or *λ_ji_* = β_0*j*_ + β_1*j*_ × DBH*_ij_* +β_2_ × Con*_ij_* + β_3_ × HetAM*_ij_* + β_4_ × HetEcM*_ij_* + β_5_ × HetErM*_ijj_* + *Φ_k_* (4)

where *p_ij_* is the predicted survival probability for each individual *i* from species *j*, and *λ_ij_* is the RGR for each individual *i* from species *j*. The parameter β_0*j*_ represents the intercept, β_1*j*_ represents the effect of the plant initial size (DBH); β_2_, β_3_, β_4_ and β_5_ represent the effect of four scale-dependent neighborhood factors; *Φ_k_* represents the random effect of the quadrat.

We chose 5 m as the minimum neighborhood radius and 30 m as the maximum neighborhood radius based on previous studies [57,58,59]. We ran the model with 26 different neighborhood radii with a spatial resolution of 1 m (i.e., 5, 6, 7, ..., 30 m from the focal individual). The Akaike’s Information Criterion (AIC) value was used to select the best-fit model across a neighborhood radius of 5 to 30 m [60]. The models with the lowest AIC values were given in the main text, and models with a neighborhood radius of 5 to 30 m are shown in Figures S3 and S4. To account for boundary effects, we excluded trees within 30 m of the plot boundary.

From the data of 255 quadrats (20 m × 20 m subplot) in this 9 ha plot, we calculated the ratio of BA of EcM species to BA of AM species (R_EA_), Shannon–Weiner index (*H*), average RGR, total BA and increment of total BA. Generalized least squares (GLS) models with certain spatial correlation structures were used to eliminate possible influences of spatial autocorrelation [61]. Due to the relatively small proportion of ErM species in the community total BA, we mainly analyzed the effects of AM and EcM tree species on species diversity and average RGR. Due to the nonlinear effect of R_EA_ on species diversity and average RGR, a nonlinear fitting method was used in the GLS model (Figure S5 and Table S3).

All analyses were conducted in R 4.1.3 [62] using the lme4 [63], lmerTest [64] and nlme packages [65].

## 3. Results

### 3.1. Neighbor Effects on All Individuals across Tree Life Stages

**Figure 2:**
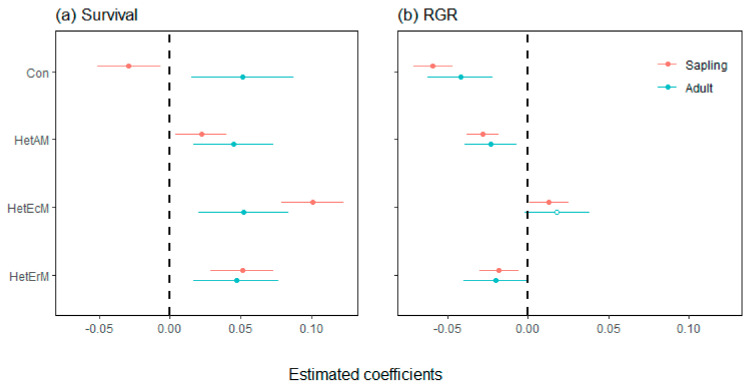
Coefficient estimates (±2SE) of neighborhood factors on survival (**a**) and RGR (**b**) of all individuals at the sapling and adult tree life stages. Solid circles indicate significant effects (*p* < 0.05), while open circles indicate non-significant effects. RGR, relative growth rates; Con, conspecific density; HetAM, heterospecific AM density; HetEcM, heterospecific EcM density; HetErM, heterospecific ErM density.

We found that CNDD had significant effects on both sapling survival and RGR. The negative effects of conspecific neighbors decreased with increasing tree life stage, turning into positive effects on the survival of adults. Different mycorrhizal neighbors showed quite similar positive effects on individual survival at both the sapling and adult stages. Specifically, compared to other heterospecific neighbors, HetAM showed a greater positive effect on adults and HetEcM showed a greater positive effect on saplings (Figure 2a). However, these heterospecific neighborhood factors had a greater negative impact on the RGR of individuals (Figure 2b). Both HetAM and HetErM negatively affected the RGR of individuals at the sapling and adult stages. Whereas HetEcM showed a positive effect on saplings, but a non-significant positive effect on adults.

### 3.2. Neighbor Effects on Different Tree Mycorrhizal Types

**Figure 3:**
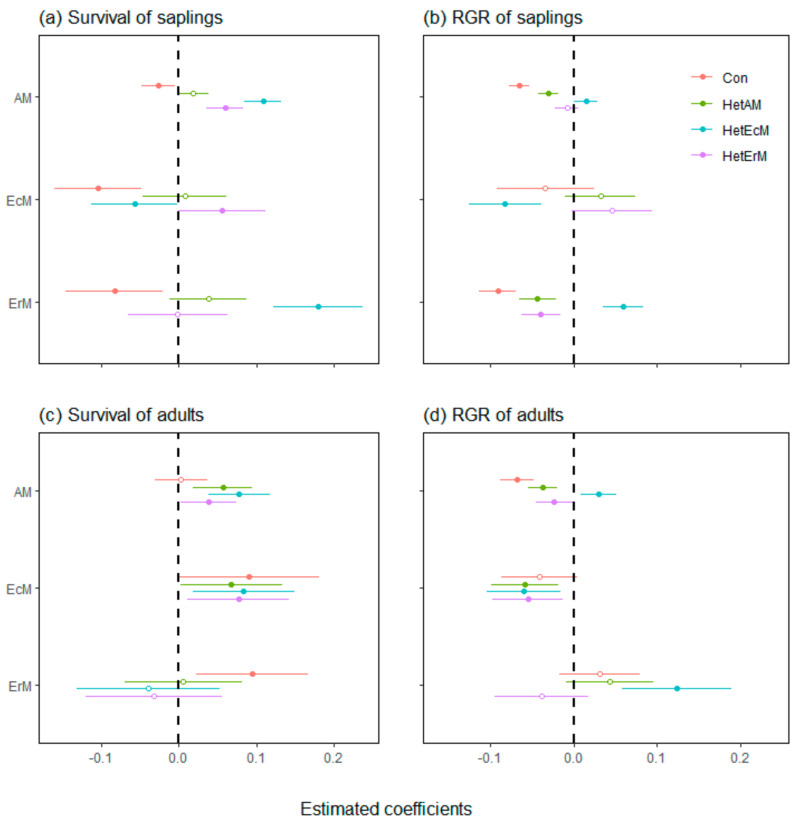
Estimated effects (±2SE) of neighborhood factors on survival and RGR of species mycorrhizal types at the sapling and adult tree life stages. Solid circles indicate significant effects (*p* < 0.05), while open circles indicate non-significant effects. RGR, relative growth rates; Con, conspecific density; HetAM, heterospecific AM density; HetEcM, heterospecific EcM density; HetErM, heterospecific ErM density.

At the sapling stage, the survival of different tree mycorrhizal types experienced strong CNDD (Figure 3a). The CNDD of EcM saplings was the strongest, followed by ErM saplings, and the CNDD of AM saplings was relatively weak. AM and ErM saplings were positively affected by HetEcM. Furthermore, AM and EcM saplings were positively affected by HetErM, while EcM saplings were negatively affected by HetEcM (Figure 3a). Compared with individual survival, the neighbor effect had a certain difference in the RGR of individuals. AM and ErM saplings were still negatively affected by Con while positively affected by HetEcM. These mycorrhizal saplings were all negatively affected by heterospecific neighbors with the same mycorrhizal type. Furthermore, ErM saplings were also negatively affected by HetAM (Figure 3b).

During the adult stage, many neighbor effects showed positive effects on the survival of different tree mycorrhizal types. Specifically, Con shifted to positive effects on EcM and ErM adults. HetAM, HetEcM and HetErM positively affected AM and EcM adults (Figure 3c). The neighbor effect of AM adults RGR was quite similar to that of saplings, with only HetErM having a significant negative effect on AM adults. EcM adults were negatively affected by HetAM, HetEcM and HetErM, whereas ErM adults were only positively affected by HetEcM (Figure 3d).

### 3.3. Relationships between R_EA_ and Species Diversity and Average RGR

**Figure 4:**
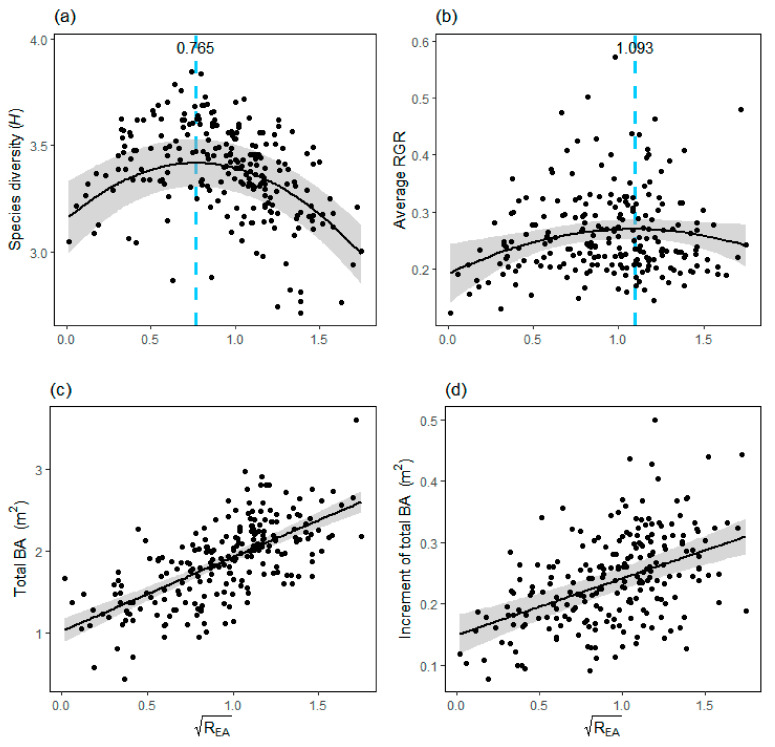
Correlations between the square root of R_EA_ with species diversity (*H*, Shannon–Weiner index) (**a**), average RGR (**b**), total BA (**c**) and increment of total BA (**d**). RGR, relative growth rates; BA, basal area; R_EA_, the ratio of BA of EcM to BA of AM tree species.

There was a nonlinearity along the square root of R_EA_ for species diversity and average RGR (Table S4). Species diversity increased rapidly as the square root of R_EA_ fell below the threshold (R_EA_ = 0.765^2^ = 0.585; Figure 4a) and turned to decrease above the threshold. The average RGR had the same pattern as species diversity, but it changed relatively slowly compared to species diversity (threshold R_EA_ = 1.093^2^ = 1.195; Figure 4b), while both total BA and the increment of total BA increased with the square root of R_EA_ (Figure 4c,d).

## 4. Discussion

CNDD varied widely with tree mycorrhizal types and tree life stages. Due to the large variances in ecological characteristics among species, the impact of different neighbors on the survival and growth of focal individuals can be complicated. The results of this study showed that separating heterospecific neighbors into distinct heterospecific mycorrhizal tree neighbors has important implications for further understanding of density-dependent effects on individuals along tree life stages. In addition, compared with other tree mycorrhizal types, the stronger CNDD of EcM tree species at the sapling stage and AM tree species at the adult stage was beneficial to the increase of species diversity. This allowed species diversity to be highest when EcM tree species had a lower proportion of BA and AM tree species had a higher proportion of BA. The EcM tree species significantly improved the survival and growth of AM and ErM tree species, which, in turn, contributed to the increase in the average RGR and total BA of the neighborhood individuals. However, when AM or EcM tree species dominated at the local scale, it reduced species diversity and average RGR. Therefore, manipulating the appropriate BA ratio of EcM to AM tree species will play an important role in maintaining biodiversity and increasing the forest carbon sink rate.

### 4.1. The Strength of CNDD Varied among Tree Mycorrhizal Types

Similar to previous studies, in the analysis of all individuals or different tree mycorrhizal types, the CNDD of sapling survival was much stronger than that of adults [56,66]. This reflected variations in CNDD along the life history. Previous studies have suggested that AM tree species usually suffer from stronger CNDD than EcM tree species [67,68,69]. Unexpectedly, the survival of EcM saplings was more negatively affected by conspecific density compared with AM and ErM saplings (Figure 3a). This may be related to the accumulation of soil pathogens [70]. In this study, the abundance of 24 EcM species only accounted for 12.70% of the entire community, but the total BA of EcM species accounted for 44.34% of the entire community, and more than half of the EcM individuals were adult trees (Table 1). This means that, compared with AM and ErM species, EcM species have a higher proportion of large trees, which may accumulate more specific pathogens and lead to higher mortality of EcM saplings. This makes it hard for EcM species to recruit saplings. The death of EcM saplings leaves space for the growth of AM and ErM saplings, thus improving species diversity. However, since surviving EcM saplings may have grown in locations with fewer pathogens, their growth was not significantly affected by CNDD.

In addition, the impacts of conspecifics on the survival and growth of AM adults tended to be more negative compared with EcM and ErM tree species (Figure 3c,d). This may be due to the lower host specificity of AM fungi, which are less able to obtain resources and resist disease than the more host-specific EcM and ErM fungi [71,72]. Since EcM and ErM adults were less likely to die from CPDD (Figure 3c), species diversity decreased with increasing EcM and ErM densities. In contrast, although the survival probability of AM adults was not affected by CNDD, the growth of AM adults decreased with increasing conspecific density. The slow growth rate of AM adults provides opportunities for the growth of EcM and ErM tree species, thereby increasing species diversity. The CNDD of EcM saplings and AM adults was stronger than that of species associated with other mycorrhizal types at the same tree life stage, indicating that tree mycorrhizal types have different contributions in maintaining species diversity along tree life stages.

### 4.2. The Different Effects of Heterospecific Mycorrhizal Type Neighbors

Consistent with most previous studies, the effect of heterospecific mycorrhizal on species, especially saplings, was weaker than that of conspecific density (Figure 2) [73]. However, different from previous studies, when the heterospecific densities were divided into different tree mycorrhizal types, the effects of these types on individual survival and growth were quite different. The positive effect of EcM density on the survival and growth of individuals of other mycorrhizal types was significantly greater than that of AM and ErM densities (Figure 3). The mantle and Hartig nets formed by EcM fungi in roots and antibiotic compounds produced by EcM fungi protect roots from soil-borne pathogens [34,42,74]. The existence of EcM neighbors hindered the accumulation of soil-borne pathogens in the environment, which, in turn, promoted the growth and survival of AM or ErM tree species. Previous studies have suggested that EcM tree species reduce species diversity with weak CNDD [38,75], but paid little attention to the role of EcM tree species in shaping community composition and increasing forest carbon sink rates by promoting the growth and survival of other species.

In addition, heterospecific mycorrhizal tree neighbors can promote the survival of focal individuals, while heterospecific AM and ErM neighbors hamper individual growth (Figure 2). While heterospecific AM and ErM neighbors attenuate the impact of natural enemies and increase the survival of focal individuals through herd immunity effects [39,40,41], these individuals also compete for resources with focal individuals, thereby slowing their growth.

Except for ErM adults, the growth of both saplings and adults of other tree mycorrhizal types was significantly inhibited by heterospecific neighbors associated with the same mycorrhizal fungus type (Figure 3b,d). Allsopp and Stock [76] also found that, with the increase of conspecific density, the mass of mycorrhizal plants decreased more rapidly than that of non-mycorrhizal plants. There are three possible reasons. First, the same mycorrhizal tree species obtain resources in a similar way through mycorrhiza [77], and competition for resources slows down its growth. Second, since the proportion of colonized root length increases with density of the same mycorrhizal tree species, more carbohydrates are transporting from the host plant to mycorrhizal fungi, which slows the growth rate of the host plant [78,79,80]. Third, there are common pathogens that infest species related to the same mycorrhizal type [21,67,81]. When the stem density of the same mycorrhizal species increased, the content of such pathogens in the neighborhood also gradually accumulated, which affected the growth of focal individuals.

### 4.3. The R_EA_ Affects Species Diversity and Forest Carbon Sink Rates

These tree mycorrhizal types play different roles in the process of community assembly. This study found that species diversity was lower when AM or EcM tree species predominated. Species diversity reached its highest value only when R_EA_ reached 0.585 (this number may vary by region or time) (Figure 4a). Compared with EcM tree species, AM tree species can indeed maintain higher species diversity at larger BA ratios. This is partially consistent with the previous studies [38,69]. This may be related to the stronger CNDD effect on AM adults and more species in AM tree types. However, the diversity decreased when the BA of AM or EcM tree species continued to increase. Carteron et al. [82] also found relatively low species diversity in forests dominated by AM or EcM tree species in the U.S. Since adults occupy more space, an increase in adults that are more likely to survive with weaker CNDD will reduce the total number of individuals in the area, leading to a reduction in species diversity.

Similar to the above results, the average RGR reached the highest value when the R_EA_ was 1.195 (Figure 4b). The results indicated that EcM tree species had a greater ability to promote the growth of surrounding individuals than AM tree species, so EcM tree species were more conducive to the improvement of forest carbon sink rates. Van Der Heijden and Horton [72] found that the EcM mycorrhizal network exchanged resources more efficiently, so EcM fungi were more able to promote seedling growth than AM fungi. However, the average RGR decreased when the BA of EcM tree species was too high. This is consistent with the above results of this study (Figure 3b,d). The RGR of EcM species decreased with increasing heterospecific EcM neighbors. When the BA of EcM tree species is higher, EcM individuals with slower RGR will reduce the average RGR of all individuals. However, we found that total BA and the increment of total BA increased with increasing R_EA_ (Figure 4c,d). This means that EcM species play a relatively important role in the increment of forest carbon sink, especially in the acceleration of forest carbon sink rates. Since there were great differences in the effects of EcM and AM tree species on species diversity or average RGR, finding the optimal proportion of EcM and AM tree species for local-scale assemblages will be important for biodiversity conservation and the increment of forest carbon sink rates.

## 5. Conclusions

The strength of CNDD varied with tree mycorrhizal types, which had different regulatory effects on species diversity at different tree life stages. EcM species significantly increased the survival and growth of AM and ErM tree species, as well as the growth and total BA of surrounding individuals. However, when AM or EcM tree species predominated, species diversity is suppressed and forest carbon sink rates are lowered. Therefore, in the context of global change, manipulating the appropriate BA ratios of AM and EcM tree species will play an important role in maintaining species diversity and increasing forest carbon sink rates. However, due to environmental differences, the optimal BA ratio of AM and EcM tree species will vary with latitude or forest type, and further exploration and research are needed.

## Figures and Tables

**Table 1:** Summary information on species mycorrhizal types.

Mycorrhizal Types	Richness	No. Saplings	No. Adults	Survival Rate	Relative Abundance	Relative Basal Area
AM	146	21,623	9932	85.26%	68.78%	49.06%
EcM	24	2721	3104	84.22%	12.70%	44.34%
ErM	9	6667	1836	92.07%	18.53%	6.60%

## Data Availability

Not applicable.

## Supplementary Materials

The following supporting information can be downloaded at: https://www.mdpi.com/article/10.3390/plants11182340/s1, Figure S1: Log-likelihood as a function of exponent *c* at 5–30 m across all individuals at sapling and adult life stages; Figure S2: Log-likelihood as a function of exponent *c* at 5–30 m in different tree mycorrhizal types at sapling and adult life stages; Figure S3: Estimates (±2SE) of neighborhood factors on survival (a) and RGR (b) of all individuals at sapling and adult life stages at 5–30 m; Figure S4: Estimated effects (±2SE) of neighborhood factors on survival and RGR of tree mycorrhizal types at sapling and adult life stages at 5–30 m; Figure S5: Correlations between BA of EcM species (BA_EcM_) and BA of AM species (BA_AM_) with species diversity (*H*, Shannon–Weiner index) (a and e), average RGR (b and f), total BA (c and g) and increment of total BA (d and h); Table S1: 179 focal species used in the analysis of neighborhood effects on survival and growth; Table S2: Optimal scales and *c* for survival and relative growth rate (RGR) of all individuals and tree mycorrhizal types at sapling and adult life stages at 5–30 m; Table S3: Coefficient estimates of the correlation between BA_EcM_ and BA_AM_ with species diversity, average RGR, total BA and increment of total BA; Table S4: Coefficient estimates of the correlation between the square root of R_EA_ (√R_EA_) with species diversity, average RGR, total BA and increment of total BA.
