# Supplementary material for: Differences in Density Dependence among Tree Mycorrhizal Types Affect Tree Species Diversity and Relative Growth Rates
Source: Plants (Basel). 2022 Sep 7;11(18):2340. doi: 10.3390/plants11182340 (PMC9505969; doi:10.3390/plants11182340)
Supplement: Supplementary file 1 [file plants-11-02340-s001.zip › plants-1883552-supplementary.pdf]

Figure S1. Log-likelihood as a function of exponent *c* at 5-30 m across all individuals at sapling and adult life stages. Each point on $curve_{mean}$ (black point) is the average value of the log-likelihood at 5-30 m at a certain *c*.

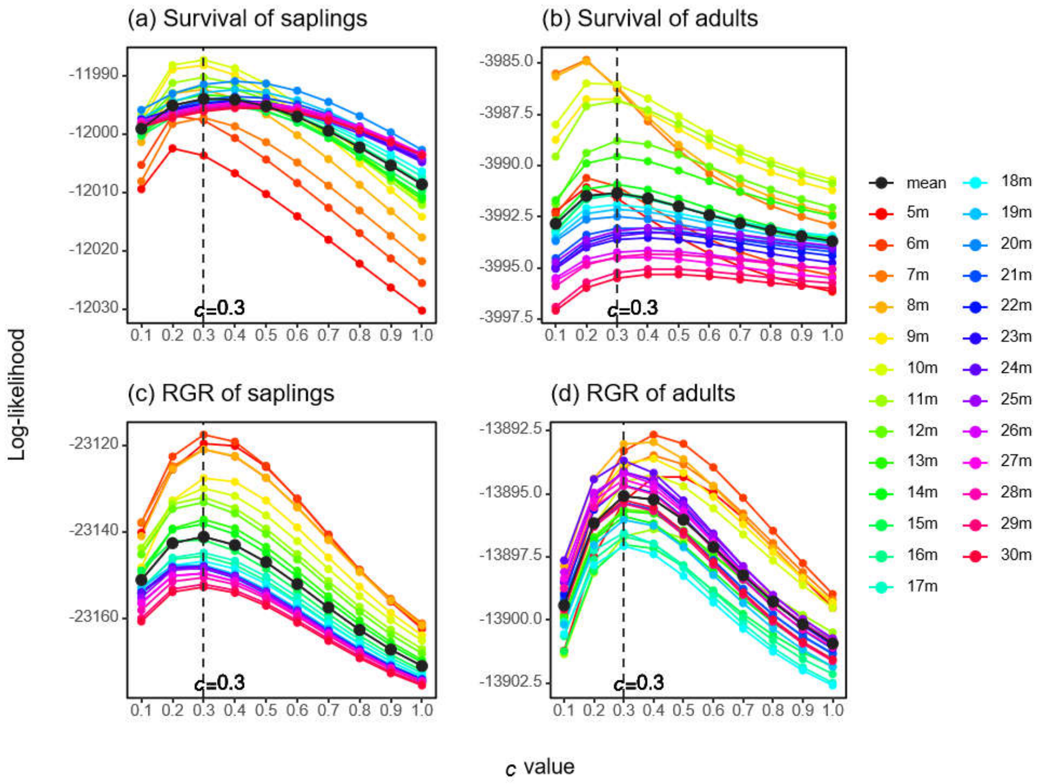

Figure S2. Log-likelihood as a function of exponent *c* at 5-30 m in different tree mycorrhizal types at sapling and adult life stages. Each point on $curve_{mean}$ (black point) is the average value of the log-likelihood at 5-30 m at a certain *c*.

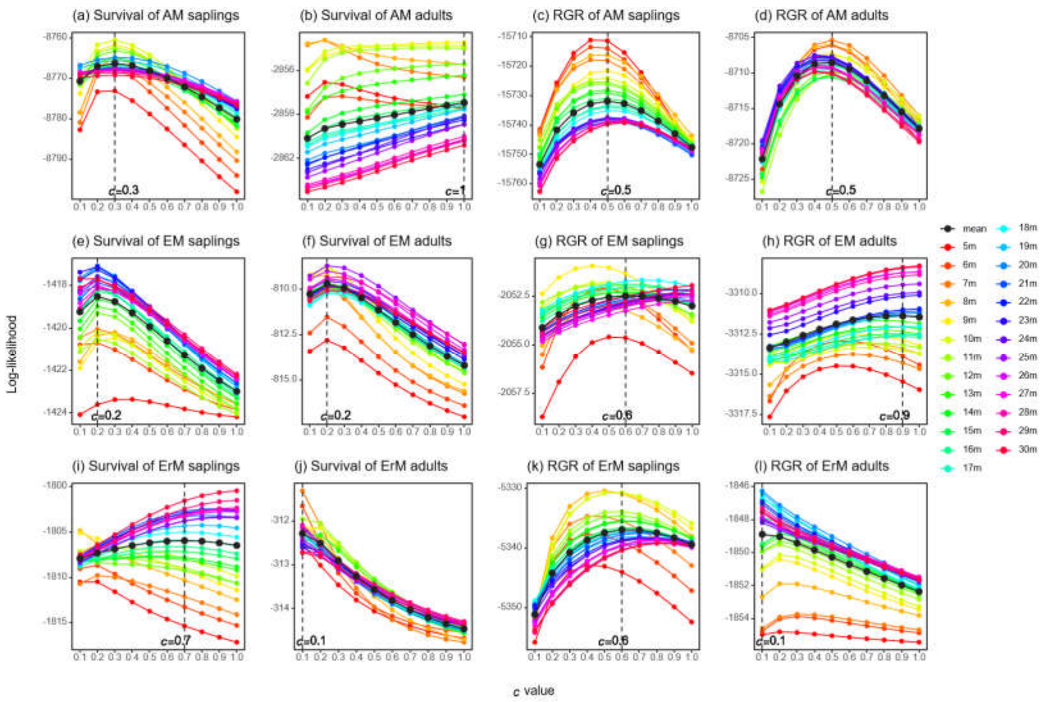

Figure S3. Estimates (± 2SE) of neighborhood factors on survival (a) and RGR (b) of all individuals at sapling and adult life stages at 5-30 m. Solid circles indicate significant effects (*P* < 0.05), while open circles indicate non-significant effects. Con, conspecific density; HetAM, heterospecific AM density; HetEcM, heterospecific EcM density; HetErM, heterospecific ErM density.

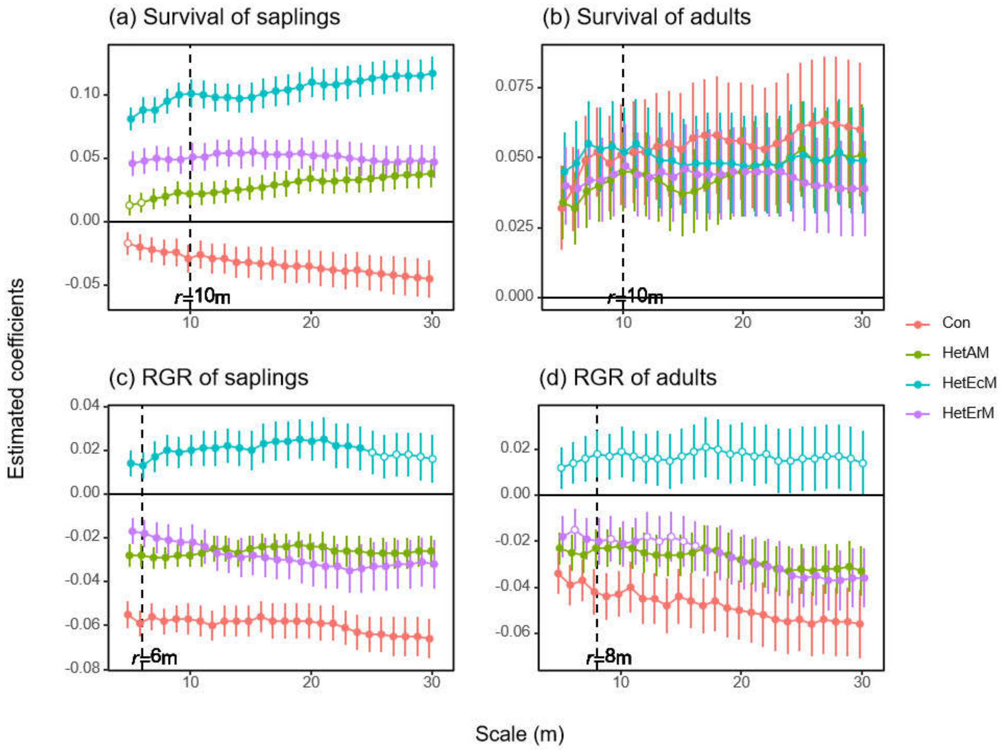

Figure S4. Estimated effects (± 2SE) of neighborhood factors on survival and RGR of tree mycorrhizal types at sapling and adult life stages at 5-30 m. Solid circles indicate significant effects (P < 0.05), while open circles indicate non-significant effects. Con, conspecific density; HetAM, heterospecific AM density; HetEcM, heterospecific EcM density; HetErM, heterospecific ErM density.

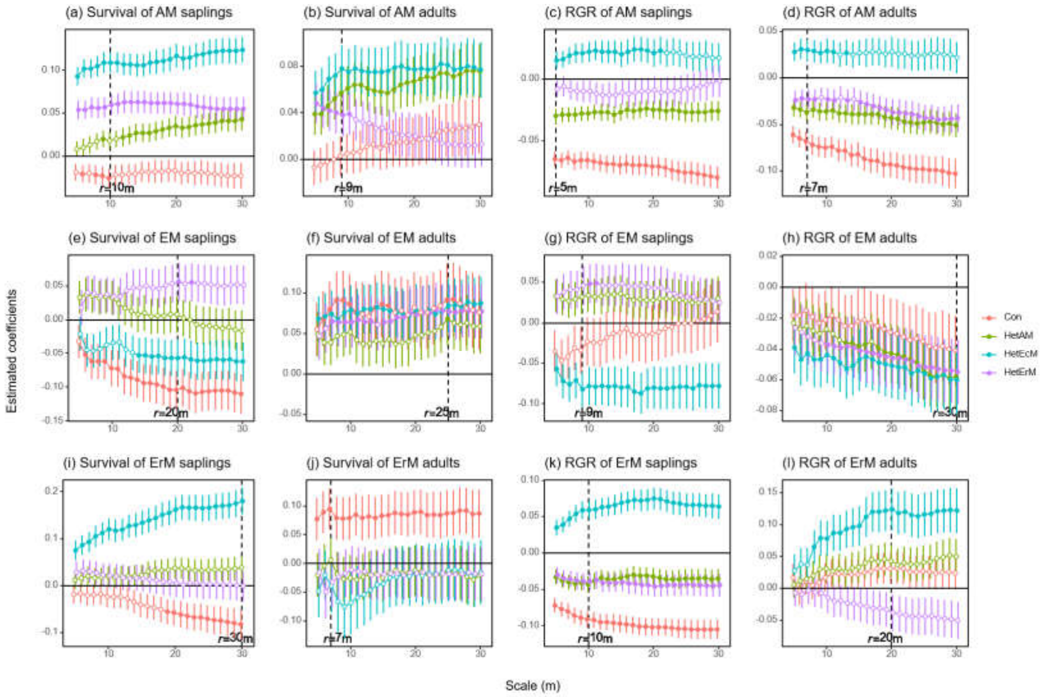

Figure S5. Correlations between BA of EcM species (BA_{EcM}) and BA of AM species (BA_{AM}) with species diversity (H, Shannon-Weiner index) (a & e), average RGR (b & f), total BA (c & g) and increment of total BA (d & h).

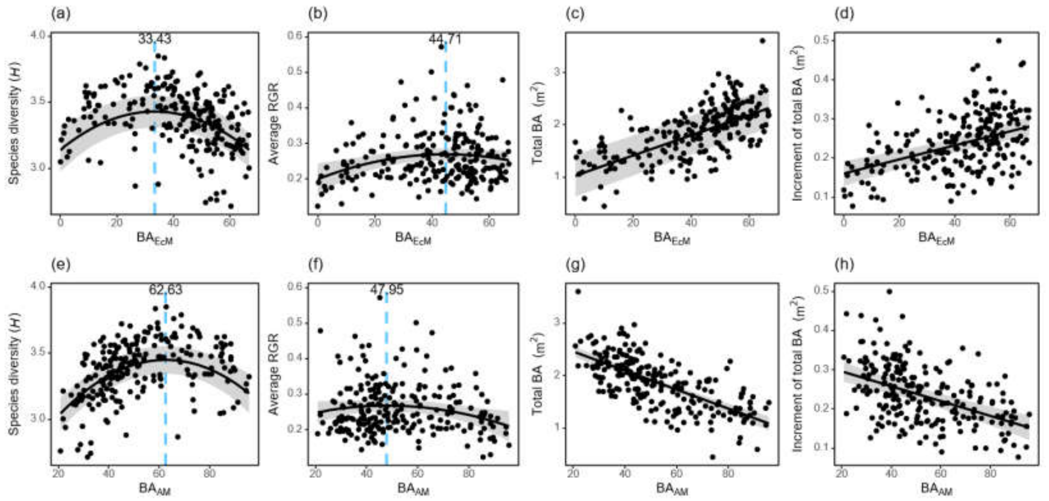

Table S1. 179 focal species used in the analysis of neighborhood effects on survival and growth. (Life form: C=canopy tree species, U=understory tree species, and S=shrub species) in subtropical evergreen broad-leaved forest in Wuyanling National Natural Reserve, Zhejiang, China.

Species	Family	Life form	Mycorrhizal type	No. individuals
Acer amplum	Sapindaceae	C	AM	26
Acer cordatum	Sapindaceae	C	AM	129
Acer davidii	Sapindaceae	C	AM	65
Acer elegantulum	Sapindaceae	C	AM	64
Acer wilsonii	Sapindaceae	C	AM	26
Adinandra glischroloma var. *macrosepala*	Pentaphylacaceae	S	AM	178
Albizia kalkora	Fabaceae	C	AM	3
Alniphyllum fortunei	Styracaceae	C	AM	263
Betula luminifera	Betulaceae	C	EcM	8
Camellia chekiangoleosa	Theaceae	U	AM	920
Camellia cuspidata	Theaceae	S	AM	3225
Carpinus polyneura	Betulaceae	C	EcM	3
Carpinus viminea	Betulaceae	C	EcM	426
Castanopsis eyrei	Fagaceae	C	EcM	1291
Castanopsis fabri	Fagaceae	C	EcM	55
Castanopsis fargesii	Fagaceae	C	EcM	1
Castanopsis tibetana	Fagaceae	C	EcM	11
Celtis biondii	Cannabaceae	C	AM	6
Choerospondias axillaris	Anacardiaceae	C	AM	7
Cinnamomum austrosinense	Lauraceae	C	AM	1
Cinnamomum japonicum	Lauraceae	C	AM	25
Cinnamomum subavenium	Lauraceae	C	AM	385
Clerodendrum cyrtophyllum	Lamiaceae	U	AM	2
Clerodendrum kaichianum	Lamiaceae	U	AM	2
Clethra barbinervis	Clethraceae	U	AM	12
Clethra delavayi	Clethraceae	U	AM	3
Cleyera japonica	Pentaphylacaceae	U	AM	520
Cleyera pachyphylla	Pentaphylacaceae	U	AM	2
Cornus controversa	Cornaceae	C	AM	2
Cornus hongkongensis subsp. elegans	Cornaceae	U	AM	556
Cryptomeria japonica var. *sinensis*	Cupressaceae	C	AM	5
Cunninghamia lanceolata	Cupressaceae	C	AM	195
Cyclocarya paliurus	Juglandaceae	C	AM	18
Dalbergia hupeana	Fabaceae	C	AM	7
Daphniphyllum macropodum	Daphniphyllac	U	AM	32

	eae			
Daphniphyllum oldhamii	Daphniphyllaceae	C	AM	76
Dendropanax dentiger	Araliaceae	U	AM	496
Diospyros japonica	Ebenaceae	C	AM	9
Diospyros morrisiana	Ebenaceae	C	AM	1
Diospyros tsangii	Ebenaceae	U	AM	8
emmenopterys henryi	Rubiaceae	C	AM	1
Elaeagnus glabra	Elaeagnaceae	S	AM	1
Elaeocarpus decipiens	Elaeocarpaceae	C	AM	4
Elaeocarpus japonicus	Elaeocarpaceae	C	AM	120
Erythroxylum sinense	Erythroxylaceae	U	AM	5
Euonymus laxiflorus	Celastraceae	S	AM	1
Euonymus myrianthus	Celastraceae	U	AM	35
Euonymus nitidus	Celastraceae	S	AM	7
Eurya alata	Pentaphylacaceae	S	AM	15
Eurya hebeclados	Pentaphylacaceae	U	AM	6
Eurya loquaiana	Pentaphylacaceae	U	AM	315
Eurya loquaiana var. *aureopunctata*	Pentaphylacaceae	U	AM	42
Eurya muricata	Pentaphylacaceae	S	AM	1355
Eurya nitida	Pentaphylacaceae	S	AM	6
Eurya rubiginosa var. *attenuata*	Pentaphylacaceae	S	AM	4226
Fagus engleriana	Fagaceae	C	EcM	72
Fagus lucida	Fagaceae	C	EcM	13
Ficus heteromorpha	Moraceae	U	AM	2
Fraxinus chinensis	Oleaceae	C	AM	9
Fraxinus insularis	Oleaceae	C	AM	3
Gardenia jasminoides	Rubiaceae	S	AM	40
Halesia macgregorii	Styracaceae	C	AM	363
Hovenia trichocarpa var. *robusta*	Rhamnaceae	C	AM	10
Hydrangea chinensis	Hydrangeaceae	S	AM	7
Hydrangea strigosa	Hydrangeaceae	S	AM	1
Idesia polycarpa	Salicaceae	C	AM	8
Ilex buergeri	Aquifoliaceae	U	AM	25

Ilex chinensis	Aquifoliaceae	C	AM	40
Ilex crenata	Aquifoliaceae	S	AM	3
Ilex elmerrilliana	Aquifoliaceae	U	AM	7
Ilex ficoidea	Aquifoliaceae	C	AM	839
Ilex formosana	Aquifoliaceae	C	AM	31
Ilex kwangtungensis	Aquifoliaceae	U	AM	75
Ilex latifolia	Aquifoliaceae	C	AM	34
Ilex litseifolia	Aquifoliaceae	U	AM	758
Ilex lohfauensis	Aquifoliaceae	U	AM	3
Ilex micrococca	Aquifoliaceae	C	AM	36
Ilex nitidissima	Aquifoliaceae	U	AM	3
Ilex rotunda	Aquifoliaceae	C	AM	52
Ilex suaveolens	Aquifoliaceae	C	AM	149
Ilex triflora	Aquifoliaceae	U	AM	1
Ilex triflora var. *kanehirai*	Aquifoliaceae	U	AM	26
Ilex wilsonii	Aquifoliaceae	C	AM	968
Illicium lanceolatum	Schisandraceae	U	AM	3
Itea omeiensis	Iteaceae	S	AM	567
Lagerstroemia subcostata	Lythraceae	C	AM	7
Lasianthus japonicus	Rubiaceae	S	AM	4
Ligustrum leucanthum	Oleaceae	U	AM	2
Lindera aggregata	Lauraceae	S	AM	448
Lindera erythrocarpa	Lauraceae	U	AM	2
Liquidambar acalycina	Altingiaceae	C	AM	153
Lithocarpus brevicaudatus	Fagaceae	C	EcM	473
Lithocarpus hancei	Fagaceae	C	EcM	50
Lithocarpus iteaphyllus	Fagaceae	C	EcM	528
Lithocarpus litseifolius	Fagaceae	C	EcM	65
Litsea elongata	Lauraceae	C	AM	119
Litsea elongata var. *faberi*	Lauraceae	C	AM	550
Lyonia ovalifolia var. *hebecarpa*	Ericaceae	U	ErM	1
Machilus leptophylla	Lauraceae	C	AM	16
Machilus litseifolia	Lauraceae	C	AM	21
Machilus pauhoi	Lauraceae	C	AM	9
Machilus phoenicis	Lauraceae	U	AM	1660
Machilus thunbergii	Lauraceae	C	AM	1262
Mallotus tenuifolius	Euphorbiaceae	U	AM	1
Malus leiocalyca	Rosaceae	C	AM	59
Manglietia yuyuanensis	Magnoliaceae	C	AM	311
Meliosma flexuosa	Sabiaceae	U	AM	59
Meliosma myriantha var. *discolor*	Sabiaceae	C	AM	16
Meliosma oldhamii	Sabiaceae	C	AM	451
Metasequoia glyptostroboides	Cupressaceae	C	AM	2
Michelia maudiae	Magnoliaceae	C	AM	491

Microtropis fokienensis	Celastraceae	S	AM	6
Morella rubra	Myricaceae	C	AM	21
Neolitsea aurata var. *chekiangensis*	Lauraceae	U	AM	2368
Neolitsea aurata var. *paraciculata*	Lauraceae	U	AM	90
Neolitsea aurata var. *undulatula*	Lauraceae	U	AM	110
Nyssa sinensis	Nyssaceae	C	AM	229
Osmanthus austrozhejiangensis	Oleaceae	U	AM	314
Osmanthus matsumuranus	Oleaceae	U	AM	3
Parakmeria lotungensis	Magnoliaceae	C	AM	4
Phoebe sheareri	Lauraceae	C	AM	24
Photinia beauverdiana	Rosaceae	U	AM	66
Photinia glabra	Rosaceae	U	AM	943
Photinia parvifolia	Rosaceae	U	AM	24
Photinia serratifolia	Rosaceae	U	AM	7
Photinia villosa	Rosaceae	U	AM	48
Picrasma quassioides	Simaroubaceae	C	AM	3
Pieris japonica	Ericaceae	U	ErM	109
Pinus hwangshanensis	Pinaceae	C	EcM	12
Pinus massoniana	Pinaceae	C	EcM	3
Pittosporum illicioides	Pittosporaceae	S	AM	5
Platycarya strobilacea	Juglandaceae	U	EcM	2
Prunus conradinae	Rosaceae	C	AM	3
Prunus phaeosticta	Rosaceae	U	AM	9
Prunus schneideriana	Rosaceae	U	AM	115
Prunus spinulosa	Rosaceae	C	AM	12
Quercus engleriana	Fagaceae	U	EcM	237
Quercus glauca	Fagaceae	C	EcM	52
Quercus jenseniana	Fagaceae	C	EcM	18
Quercus multinervis	Fagaceae	C	EcM	90
Quercus myrsinifolia	Fagaceae	C	EcM	268
Quercus sessilifolia	Fagaceae	C	EcM	231
Quercus shennongii	Fagaceae	C	EcM	27
Quercus stewardiana	Fagaceae	C	EcM	1889
Rhaphiolepis indica	Rosaceae	U	AM	46
Rhododendron championiae	Ericaceae	U	ErM	85
Rhododendron latoucheae	Ericaceae	U	ErM	4105
Rhododendron mariesii	Ericaceae	U	ErM	1255
Rhododendron ovatum	Ericaceae	U	ErM	2364
Rhododendron simsii	Ericaceae	S	ErM	138
Sassafras tzumu	Lauraceae	C	AM	75
Schima superba	Theaceae	C	AM	1085
Schoepfia jasminodora	Schoepfiaceae	U	AM	33
Semiliquidambar caudata	Altingiaceae	C	AM	1
Sorbus folgneri	Rosaceae	C	AM	2

Styrax dasyanthus	Styracaceae	U	AM	31
Styrax odoratissimus	Styracaceae	U	AM	4
Styrax suberifolius	Styracaceae	C	AM	179
Symplocos anomala	Symplocaceae	U	AM	414
Symplocos botryantha	Symplocaceae	U	AM	154
Symplocos chinensis	Symplocaceae	U	AM	2
Symplocos cochinchinensis var. *laurina*	Symplocaceae	U	AM	24
Symplocos heishanensis	Symplocaceae	C	AM	137
Symplocos lancifolia	Symplocaceae	U	AM	5
Symplocos lucida	Symplocaceae	U	AM	183
Symplocos pendula var. *hirtistylis*	Symplocaceae	U	AM	7
Symplocos stellaris	Symplocaceae	U	AM	140
Symplocos sumuntia	Symplocaceae	U	AM	37
Syzygium buxifolium	Myrtaceae	U	AM	344
Taxus wallichiana var. *mairei*	Taxaceae	C	AM	2
Ternstroemia gymnanthera	Pentaphylacaceae	U	AM	838
Tetradium glabrifolium	Rutaceae	C	AM	3
Toxicodendron succedaneum	Anacardiaceae	C	AM	150
Vaccinium bracteatum	Ericaceae	U	ErM	68
Vaccinium mandarinorum	Ericaceae	S	ErM	378
Viburnum erosum	Adoxaceae	S	AM	83
Viburnum sempervirens var. *trichophorum*	Adoxaceae	S	AM	31
Viburnum sympodiale	Adoxaceae	U	AM	17
Yulania cylindrica	Magnoliaceae	C	AM	10

Table S2. Optimal scales and *c* for survival and relative growth rate (RGR) of all individuals and tree mycorrhizal types at sapling and adult life stages at 5-30m.

		c		Scale (m)	
		Sapling	Adult	Sapling	Adult
Survival	All individuals	0.3	0.3	10	10
	AM tree species	0.3	1.0	10	9
	EcM tree species	0.2	0.2	20	25
	ErM tree species	0.7	0.1	30	28
RGR	All individuals	0.3	0.3	6	8
	AM tree species	0.5	0.5	5	7
	EcM tree species	0.7	0.9	9	30
	ErM tree species	0.6	0.1	10	19

Table S3. Coefficient estimates of the correlation between BA_{EcM} and BA_{AM} with species diversity, average RGR, total BA and increment of total BA. BA, basal area; BA_{EcM}, BA of EcM tree species; BA_{AM}, BA of AM tree species; RGR, relative growth rates.

Response variable	Explanatory variable	*Estimates*	*SE.*	*P*
Species diversity	BA_{EcM}	0.017	0.003	0.000
	BA_{EcM}^2	0.000	0.000	0.000
Average RGR	BA_{EcM}	0.003	0.001	0.006
	BA_{EcM}^2	0.000	0.000	0.013
Total BA	BA_{EcM}	0.019	0.002	0.000
Increment of total BA	BA_{EcM}	0.002	0.000	0.000
Species diversity	BA_{AM}	0.029	0.003	0.000
	BA_{AM}^2	0.000	0.000	0.000
Average RGR	BA_{AM}	0.002	0.001	0.082
	BA_{AM}^2	0.000	0.000	0.040
Total BA	BA_{AM}	-0.018	0.001	0.000
Increment of total BA	BA_{AM}	-0.002	0.000	0.000

Table S4. Coefficient estimates of the correlation between the square root of R_{EA} ($\sqrt{R_{EA}}$) with species diversity, average RGR, total BA and increment of total BA. RGR, relative growth rates; BA, basal area; R_{EA}, the ratio of BA of EcM to BA of AM tree species.

Response variable	Explanatory variable	*Estimates*	*SE.*	*P*
Species diversity	$\sqrt{R_{EA}}$	0.685	0.142	0.000
	R_{EA}	-0.447	0.070	0.000
Average RGR	$\sqrt{R_{EA}}$	0.143	0.055	0.009
	R_{EA}	-0.066	0.027	0.016
Total BA	R_{EA}	0.903	0.074	0.000
Increment of total BA	R_{EA}	0.092	0.014	0.000
